# Fungal and bacterial oxylipins are signals for intra- and inter-cellular communication within plant disease

**DOI:** 10.3389/fpls.2022.823233

**Published:** 2022-09-16

**Authors:** Marzia Beccaccioli, Nicoletta Pucci, Manuel Salustri, Marco Scortichini, Marco Zaccaria, Babak Momeni, Stefania Loreti, Massimo Reverberi, Valeria Scala

**Affiliations:** ^1^Department of Environmental Biology, Sapienza University of Rome, Rome, Italy; ^2^Research Centre for Plant Protection and Certification, Council for Agricultural Research and the Analysis of Agricultural Economics (CREA), Rome, Italy; ^3^Research Centre for Olive, Fruit and Citrus Crops, Council for Agricultural Research and the Analysis of Agricultural Economics (CREA), Rome, Italy; ^4^Department of Biology, Boston College, Newton, MA, United States

**Keywords:** lipids, oxylipins, *Xylella fastidiosa*, *Fusarium* spp, *Aspergillus* spp, *Olea europaea L*., *Zea mays (L)*

## Abstract

Lipids are central at various stages of host–pathogen interactions in determining virulence and modulating plant defense. Free fatty acids may act as substrates for oxidizing enzymes [e.g., lipoxygenases (LOXs) and dioxygenases (DOXs)] that synthesize oxylipins. Fatty acids and oxylipins function as modulators of several pathways in cell-to-cell communication; their structural similarity among plant, fungal, and bacterial taxa suggests potential in cross-kingdom communication. We provide a prospect of the known role of fatty acids and oxylipins in fungi and bacteria during plant–pathogen interactions. In the pathogens, oxylipin-mediated signaling pathways are crucial both in development and host infection. Here, we report on case studies suggesting that oxylipins derived from oleic, linoleic, and linolenic acids are crucial in modulating the pathogenic lifestyle in the host plant. Intriguingly, overlapping (fungi-plant/bacteria-plant) results suggest that different inter-kingdom pathosystems use similar lipid signals to reshape the lifestyle of the contenders and occasionally determine the outcome of the challenge.

## Host–pathogen communication through the oxylipin language

Host–pathogen recognition relies upon an elaborate multi-molecular communication. In this context, oxylipins—oxidized fatty acids (FAs)—produced in several *phyla* can regulate numerous events associated with physiological and pathological processes (Blée, 2002; Andreou et al., 2009; Mosblech et al., 2009; Ambaw et al., 2021; Liu et al., 2021; Fernandes and Ghag, 2022). The term oxylipin (Gerwick et al., 1993) refers to a broad family of secondary metabolites originated from the oxidation of polyunsaturated and/or monounsaturated FAs (PUFAs and/or MUFAs) (Mosblech et al., 2009), which comprises a complex array of products: alcohols, aldehydes, ketones, acids, and hydrocarbon gases, generated *via* enzymatic and non-enzymatic processes. Oxylipins share significant structural and functional similarities across different mammal, plant, fungal, and bacterial taxa, including part of their biosynthetic pathway, structure, function, and modifications.

In plant, oxylipins are signaling molecules involved in the regulation of development and immunity. Jasmonates are the better-characterized oxylipins in plants and include jasmonic acid (JA), its precursor 12-oxo-phytodienoic acid (12-OPDA), and JA derivatives such as methyl jasmonate and JA-isoleucine. JA and its derivatives act as defense regulators, influencing the reproductive and pathogenetic processes during the interaction with both beneficial and pathogenic microorganisms (Andreou et al., 2009; Deboever et al., 2020); pathogen infection and pest wounding are the best-studied environmental triggers, in this context (Farmer et al., 2003; Block et al., 2005; Gorman et al., 2021; Shaban et al., 2021). The well-characterized JA pathway activation starts with conjugated lipids in the chloroplast membranes (e.g., monogalactosyldiacylglycerol). Lipase A1 mediates the release of α-linolenic acid, and LOX enzyme catalyzes PUFA dioxygenation. Oxygenation may happen at several positions along the carbon chain. For JA, important PUFA precursors are α-linolenic acid (Wasternack and Strnad, 2018) or, alternatively, the hexadecatrienoic acid (Chini et al., 2018). Local defenses and systemic acquired resistances (e.g., SAR, ISR) require JA involvement (Ryan and Moura, 2002; Yu et al., 2022) to express a distinct set of defense-related genes (Okada et al., 2015). Biosynthesis of other plant oxylipins is initiated by the 9 and 13-LOX and α-dioxygenase (α-DOX), or monooxygenases, which all catalyze the oxygenation of linoleic acid and linolenic acid (mainly) into reactive hydroperoxides, followed by a secondary modification by cytochrome P450 enzymes or peroxygenases (Blée, 2002; Hamberg et al., 2003; Andreou and Feussner, 2009). Oxylipins can also be produced nonenzymatically through free radical-mediated oxygenation (Oenel et al., 2017). Plant (or phyto-) oxylipins are produced under abiotic and biotic stress, as well as during beneficial interactions (Blée, 2002; Feussner and Wasternack, 2002; Prost et al., 2005). Their role spans from antimicrobial agents to signaling molecules. Recently, the employment of oxylipins as effective and commercial antimicrobial agents has been discussed (Deboever et al., 2020). Plants produce diversified oxylipins for different purposes, and oxylipins can act directly against the pathogen (such as *Fusarium* spp.) but also as attractors of biocontrol agents (such as *Trichoderma* spp.) (Gol et al., 2018; Lombardi et al., 2018; Wang et al., 2020). As signaling molecules, they have been implicated in several pathways, including morphogenesis, and as determinants of the hypersensitive response (HR) in incompatible plant–pathogen interactions (Mehta et al., 2021).

In fungi and bacteria, oxylipins control lifestyle and quorum sensing *inter alia* (Su et al., 1995; Tsitsigiannis et al., 2005b). The “oxylipin regulation system” in fungi and bacteria can employ host oxylipins to improve virulence (e.g., enhancing toxin production or switching to biofilm stage) (Burow et al., 2000; Brodhagen et al., 2008; Martínez et al., 2019) and affect reproduction rate within host tissues by increasing the sporulation (Scarpari et al., 2014). Bacterial oxylipins impact the plant defense mechanisms, as in the case of coronatine production in *Pseudomonas syringae*, a toxin that mimics the plant hormone JA-isoleucine with the aim to induce the opening of stomata to enable bacterial ingress (Zheng et al., 2012). These features support the hypothesis that oxylipins may act as “words” in the lipid common language in host–pathogen communication with a paracrine activity, as suggested by Niu and colleagues (Tsitsigiannis and Keller, 2007; Christensen and Kolomiets, 2011; Niu et al., 2020).

Discriminating which oxylipins are produced by the host and which by the pathogen is not trivial, since the two actors will produce the same molecules at the same time. Oxylipins play a relevant ecological role for the producers (i.e., plants, fungi, and bacteria) and their interspecific interactions (Siebers et al., 2016; Beccaccioli et al., 2021b). In plant–pathogen interactions, plants (the host) produce oxylipins to systemically signal pathogen attack, mounting an efficient defense system and interfering with pathogen growth and reproduction (Burow and Nesbitt, 1997; Brodhagen et al., 2008; Andreou et al., 2009; Scarpari et al., 2014).

Numerous evidence shows that oxylipins mediate interspecies signaling among eukaryotes (Pohl and Kock, 2014). *Trichoderma virens* stimulates symbiont-induced systemic resistance in maize by promoting the release of 12-OPDA and α-ketol of octadecadienoic in the xylem sap (Wang et al., 2020). In plants, 9-HPODE and 13-HPODE alter the secondary metabolism and conidiation in *Aspergillus flavus* (Calvo et al., 1999), *Colletotrichum graminicola* (Gorman et al., 2021), *Verticillium dahliae* (Shaban et al., 2021), *Fusarium oxysporum* (Fernandes and Ghag, 2022), and *inter alia*. Fungal oxylipins are involved in the control of sexual and asexual structures formation, secondary metabolism, density-dependent growth, and in the interaction with hosts (Reverberi et al., 2010; Brodhun and Feussner, 2011; Christensen and Kolomiets, 2011). In bacteria, very recent studies in *Pseudomonas aeruginosa* and *Xylella fastidiosa* suggest a role in mediating autocrine or paracrine signals in the communication with their hosts or vectors (Martínez et al., 2019; Niu et al., 2020; Scala et al., 2020).

During infection, host–pathogen communication determines the fate of the interaction. Here, we show how oxylipins represent a common language shared among plant and pathogens, both fungal and bacterial. Some case studies related to oxylipin-mediated plant–pathogen interaction regarding interaction between host plants and filamentous fungi or bacterial pathogens will be presented.

## Fungal oxylipins

From a chemical point of view, oxylipins are products of oxygenation of MUFAs or PUFAs. FAs are the main components of several complex lipids (acylglycerols, glycerophospholipids, glycolipids, sphingolipids, and sterol) from where they may be cleaved. Free FAs are diversified by length and degree of unsaturation. PUFA availability is crucial for the cell because, based on the degree of saturation, they alter cell membrane fluidity, the arrangement and availability of receptors, influencing signal transduction regulation. In this regard, fatty acids may act directly on the receptors (de Angelis et al., 2016).

Oxidation of FAs may be spontaneous in the presence of reactive oxygen species (ROS) or a consequence of enzymatic activity. The study of oxylipins in fungi followed the discovery of the role of lipid hydroperoxides in *Aspergillus parasiticus* (Fabbri et al., 1983) and of the *psi* factors (precocious sexual inducers), a series of fatty acid-derived oxylipins involved in the regulation of the development of spores and conidia (Champe et al., 1987). Different studies have elucidated the biosynthetic pathways of these molecules. Phospholipids and acylglycerides provide the substrate to oxylipin synthesis: phospholipases release oleic, linoleic, linolenic, and arachidonic acids, central elements for the formation of fungal oxylipins (Sakuradani et al., 2009; Beccaccioli et al., 2019).

**Figure 1:**
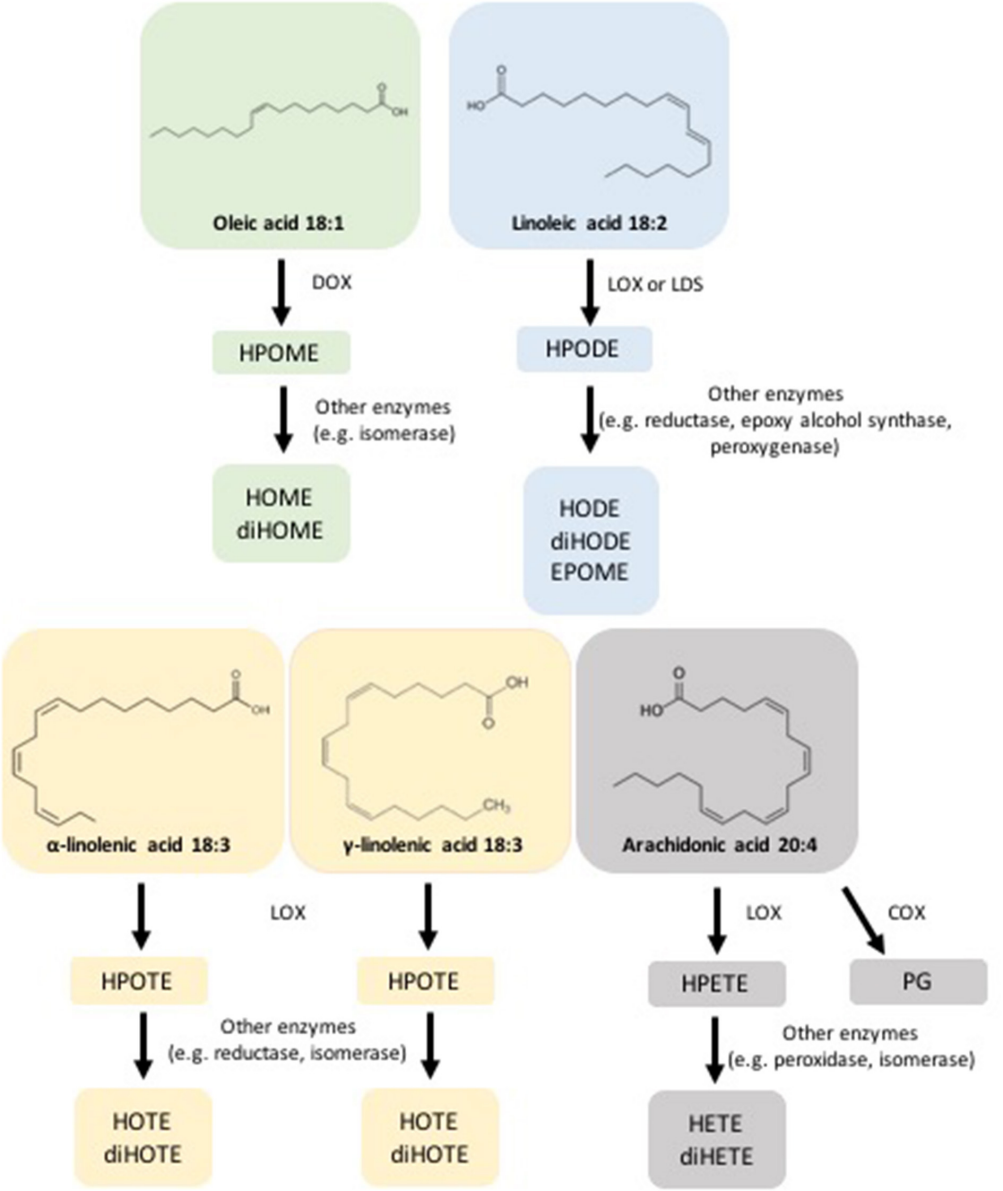
Fungal oxylipins. Oleic acid (18:1) can be oxidized by LDS enzyme and converted in hydroperoxyoctanoic acid (HPOMEs); other enzymes (e.g. isomerases) convert HPOME in hydroxyoctanoic acid (HOME), and di-hydroxyoctanoic acid (diHOME). Linoleic acid (18:2) can be oxidized by LOX enzyme and converted in hydroperoxyoctadecadienoic acid (HPODE); other enzymes (e.g. reductases) convert HPODE in hydroxyoctadecadienoic acid (HODE), and di-hydroxyoctadecadienoic acid (diHODE). Linoleic acid (18:2) is also the substrate of LDS that catalyzes the conversion in hydroperoxyoctadecadienoic acid (HPODE); other enzymes (e.g. epoxidases) convert HPODE in hydroxyoctadecadienoic acid (HODE), di-hydroxyoctadecadienoic acid (diHODE), and epoxyoctadecenoic acids (EPOME). LOX enzyme acts upon α/δ-Linolenic acid (18:3) to generate hydroperoxyoctadecatrienoic acid (HPOTE); HPOTE is the substrate of other enzymes to generate hydroxyoctadecatrienoic acid (HOTE) and di-hydroxyoctadecatrienoic acid (diHOTE). Arachidonic acid (20:4) can be oxidized by LOX and converted in hydroperoxyeicosatetraenoic acid (HPETE); HPETE is the substrate of hydroxyeicosatetraenoic acid (HETE), di-hydroxyeicosatetraenoic acid (diHETE). COX enzyme convert the arachidonic acid (20:4) in prostaglandins (PGs).

**Figure 2:**
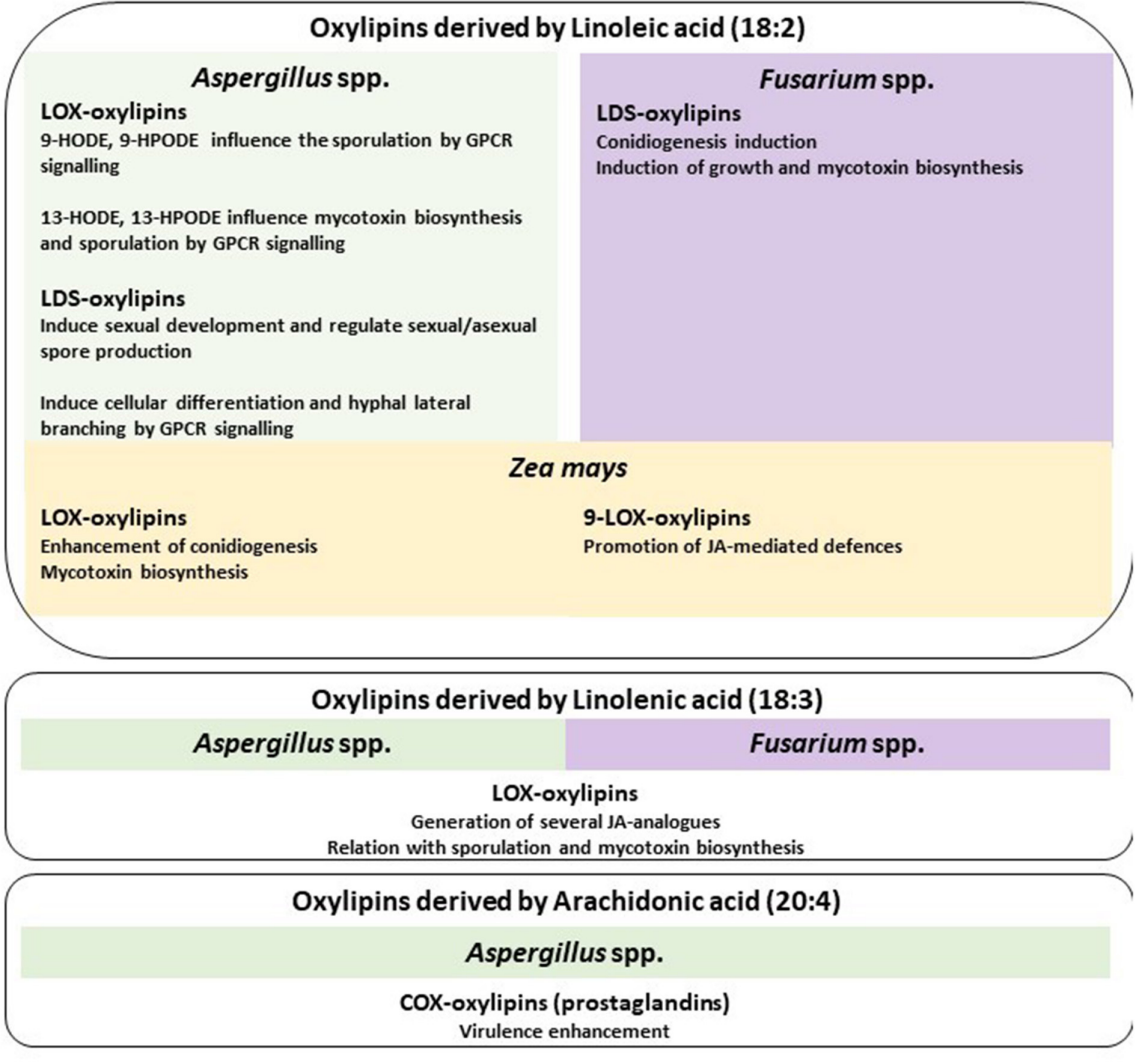
Oxylipin functions. Linoleic acid-derived oxylipins are described for *Aspergillus* spp., *Fusarium* spp., and maize. For *Aspergillus* spp., the main signaling process mediated by lipoxygenases (LOX) and linoleate diol synthases (LDS) is reported, for *Fusarium* spp. is described the LDS involvement, and for maize the LOX-mediated functions. Linolenic acid oxylipins derived from LOX activity shared the same functions in *Aspergillus* spp. and *Fusarium* spp.

The first evidence of enzymatic formation of oxylipins in fungi was found in *Gaeumannomyces graminis*, the causal agent of root and crown rot diseases. *G. graminis* produces oxylipins *via* FA oxidation with LOXs, DOXs [including the transformation of linoleic acid into dihydroxy-linoleate by 8-DOX (also named linoleate diol synthase or LDS)], and cyclooxygenase (COX) (Su et al., 2000). More recently, fatty acid dioxygenase-cytochrome P450 fusion enzymes have been identified in several pathogenic fungi, along with the implication of several oxylipins such as 8-, 9-, or 10-hydroperoxy metabolite biosynthesis (Oliw, 2021). Each fatty acid may generate different oxylipins based on enzymatic activity and spontaneous reactions available in the oxidant environment. We list known fungal oxylipins (Figure 1) and present examples of their function in relation to their chemical origin based on the class of oxygenase (LOX, DOX or LDS, and COX) (Figure 2), with a focus on the role in plant–fungus interaction.

### Enzymatic biosynthesis of oxylipins

#### LOX-derived oxylipins in fungi

LOX-mediated oxygenation may occur on linoleic or linolenic FAs. Early evidence was reported in *Aspergillus nidulans*; activity on linoleic acid generates oxylipins 9-HODE and 9-HPODE that are involved in sporulation control (Calvo et al., 1999). LOX activity on linoleic acid may also form 13-HODE and 13-HPODE, inhibiting mycotoxin production in *Aspergillus* spp. (Burow and Nesbitt, 1997) and promoting sporulation (Calvo et al., 1999). Both 9-LOX and 13-LOX, which differ in the position at which they cause oxygenation, stimulate cAMP production by fungal G protein-coupled receptor (GPCR) signaling (Affeldt et al., 2012), suggesting its activation to transduce autocrine signaling. In the mycoparasite *Trichoderma atroviride*, LOX1 is indispensable for 6-pentyl-2H-pyran-2-one production and to parasitize and antagonize host fungi, for conidiation in darkness, response to injury (i.e., 9-HODE, 13-HODE, 9-oxoOTrE, and 13-HOTrE), and production of volatile organic compounds. LOX1 in *T. atroviride* is required during the interaction with the host plant, particularly in *Arabidopsis thaliana*, to induce systemic resistance against the plant-pathogenic fungus *Botrytis cinerea* (Speckbacher et al., 2020). Fungal oxylipins derived from linolenic acid by LOX-oxidation may generate several JA analogs borrowed by the plant and act as precursors for JA, methyl-JA, and 12-OH-JA formation. These compounds were first identified in *Fusarium oxysporum* and *Aspergillus niger* (Miersch et al., 1999a,b). In plants, 12-OH-JA represents the inactive form of JA. It is likely to prevent the inhibitive effects of accumulation of JA on plant development and growth. In *Ganoderma lucidum*, the presence of 12-OH-JA also regulates host colonization by inhibiting methyl-JA formation, important for plant defense (Patkar et al., 2015). More recently, research has shown that *Magnaporthe oryzae*, the causal agent of the rice blast, can autonomously produce analogs of JA to control its pathogenic development (Liu et al., 2021).

#### DOX/LDS-derived oxylipins in fungi

Several fungi possess genes encoding for DOX which is involved in oxylipin biosynthesis. DOX enzymes contain a catalytic domain often fused to a functional cytochrome P450 at the C-terminal end. Therefore, they constitute a peculiar group within the peroxidase-cyclooxygenase superfamily. Their oxidizing activity toward linoleic acid produces five different hydroperoxides. The CYP450 domain can then transform these hydroperoxides into diols, epoxy alcohols, and allene oxides which all support sporulation, hyphal branching, and development in filamentous fungi. Fungal pathogens with biotrophic [e.g., *Blumeria graminis* (Rupasinghe et al., 2006)*, Ustilago maydis* (Huber et al., 2002)], hemibiotrophic [e.g., *Fusarium verticillioides* (Scala et al., 2014)], and necrotrophic [e.g., *Botrytis cinerea* (Niu et al., 2020)] phenotypes express these “fusion enzymes” during pathogenesis to support virulence.

LDS-derived oxylipins have different roles across fungal species. In *F. verticillioides*, linoleic acid is the substrate of LDS from which 8-HPODE and 8,13-diHODE are derived. These oxylipins can influence fungal growth and mycotoxin production (Scala et al., 2013, 2014). LDS oxylipins also promote pathogenesis and modulate the expression of maize oxylipins which, in turn, produce 9-LOX oxylipins to trigger JA-mediated defense (Battilani et al., 2018). In *A. nidulans*, 5,8-diHODE, produced from linoleic acid, regulates sexual development and the conidiation/sporulation ratio (Champe et al., 1987; Mazur et al., 1990; EbrahimáEl-Zayat, 1991; Tsitsigiannis and Keller, 2007; Brown et al., 2008). In *Aspergillus fumigatus* and *Aspergillus flavus*, 5,8-diHODE and 7,8-diHODE induce cell differentiation and lateral branching through GPCRs. Psi-producing oxidase (essentially a group of oxygenases such as LDS) is largely conserved among filamentous fungi; the synthesis of diol-containing oxylipins, primarily 5,8- and 7,8-diHODE, might represent a crucial step in the organization of fungal “morphology” (namely hyphal branching and polarity) and even “escape” from antifungals (Niu et al., 2020). For instance, in the pathogenic ascomycete *Magnaporthe grisea*, 5,8-diHODE accumulates during appressoria formation (Niu et al., 2020) paving the way for host infection. Apparently, these species of diol-oxylipins generate a signaling network in the mycelial mat to tightly adapt hyphal morphology to the environment.

#### COX-derived oxylipins in fungi

COX produces active mediators of inflammatory response from arachidonic acid oxygenation. The main findings on fungal development focus on oxylipins deriving from linoleic acid involved in sporulation and mycotoxin biosynthesis in *A. flavus, A. parasiticus* (Calvo et al., 1999), and *F. verticillioides* (Gao et al., 2007). Recently, research has also focused on fungal production of jasmonate analogs (Eng et al., 2021). Information is more scarce on oxylipins generated from arachidonic acid, a fatty acid mainly present in extremophile fungi such as *Mortierella alpina* (Kikukawa et al., 2018). In *A. fumigatus*, arachidonic acid is oxygenated by COX-like enzymes to produce prostaglandins to enhance virulence (Tsitsigiannis et al., 2005a). In *Cryptococcus neoformans* and *Candida albicans*, several studies show that fungal prostaglandins promote colonization and infection (Noverr et al., 2001, 2003).

### Oxylipin signaling in plant–fungus interactions

Mechanisms of oxylipin biosynthesis and signaling are similar in plants and fungi. Here, we report examples on how plant and fungus exploit the oxylipins to exchange messages involved in the outcome of the interaction.

The structural similarity between plant and fungal oxylipins can be exploited to reciprocally manipulate each other's signaling. In *A. flavus* on maize, thanks to this structural similarity, the pathogen's oxylipins can act on the plant's oxylipinogenic pathways, and *vice versa*. This cross talk among host and pathogen was demonstrated in different experimental settings, as reported below. In *Aspergillus* cultures, the exogenous application of plant oxylipins (i.e., 9-HPODE naturally produced by maize) increases sporulation and mycotoxin production. In *Aspergillus* deletion mutants for the DOX *ppoA* and *ppoC*, the wild-type phenotype can be restored through the insertion of maize lipoxygenase, suggesting a complementing activity. Inoculation of peanut seeds with *Aspergillus ppo*-mutants reduces LOX expression in seed, suggesting that fungal oxylipins are related to the plant *LOX* expression (Brodhagen et al., 2008).

The hydroperoxide 9-HODE generated by LOX activity offers an example of a shared signaling molecule. In plants, it induces programmed cell death, as observed in tomato protoplasts (Knight et al., 2001); in fungi, it induces sporulation and the cAMP-G protein-coupled pathway-mediated signaling (Calvo et al., 1999; Affeldt et al., 2012). Furthermore, during the interaction between maize and specific fungal pathogens, the disruption of 9-LOX from maize promotes increased resistance to the fungal pathogen and decreased mycotoxin contamination (Gao et al., 2007). 9-HODE seems to be very important for the progression of infection.

The *F. verticillioides*-maize pathosystem provides numerous examples of oxylipins as mediators of fungus–plant interaction. A close correlation exists between the fumonisin B1 accumulation and oxylipin signaling. When fumonisin is produced, maize increases the release of 9-HODE, suggesting an active role in infection development (Dall'Asta et al., 2014; Beccaccioli et al., 2021a). Further evidence shows that, in maize, the 9-LOX (*ZmLOX3*) deletion alters *F. verticillioides* fitness, decreasing conidiation and fumonisin B_1_ synthesis (Gao et al., 2007). In addition, when *FvLDS1* is deleted in the pathogen and *ZmLOX3* is mutagenized in the host, resistance to the infection increased, *ZmLOX4, ZmLOX5*, and *ZmLOX12* expression was upregulated, and the fungus showed decreased fumonisin production. The *ZmLOX4* and *ZmLOX5* mutants were more susceptible to *F. verticillioides* and showed reduced levels of JA, suggesting the relevance of JA-mediated defense signaling. Intriguingly, *F. verticillioides* infecting the *ZmLOX3* mutant is less effective at accumulating oxylipins from linoleate diol synthase and properly infecting maize kernels; this suggests that the oxylipins derived by ZmLOX3 activity are central to fungal virulence (Battilani et al., 2018).

The coexistence of a common language among hosts and pathogens supports the idea that a common receptor may also exist, and it could be represented by the GPCRs (Brown et al., 2018). Oxylipin perception has been explored only partially. In mammals, oxylipins are perceived by GPCRs (Funk, 2001; Noverr et al., 2003) in the plasma membrane (Funk, 2001). G2A is a GPCR receptor, characterized in the lymphoid tissues, and is able to recognize oxylipins derived from linoleic and arachidonic acid (Obinata et al., 2005). GPCR promotes root growth and ROS scavenging probably in the context of oxylipins pathway perception of abiotic stress in *Arabidopsis* and cotton (Lu et al., 2019). In fungi, several research studies showed the GPCR involvement in numerous functions among which oxylipin sensing (Affeldt et al., 2012). G proteins have a crucial role in sensing external ligands such as nutrients, hormones, proteins, pheromones and other peptides, ions, hydrophobic surfaces, and light (Kochman, 2014). Several works suggest that fungal GPCR-mediated signaling is linked to pathogenesis and could be considered a target for disease control (Brown et al., 2018). Secondary metabolism seems to be related to the G protein pathway as well through the activity of transcription factors related to toxin biosynthesis (Gao et al., 2021).

## Bacterial oxylipins

The “story” of oxylipins in bacteria is far more recent than in fungi. Only recently, this class of lipid compounds was found to be significant in regulating different aspects of the bacterial lifestyle. The molecules from which oxylipins originate, that is, lipids, have a quite vast background. Many lipid molecules in bacterial cells, such as hopanoids and ornitholipids, are absent in eukaryotes, while others are shared (e.g., phospholipids). Lipids play important roles in bacterial cell-to-cell communication by regulating quorum sensing (QS) and in the interactions with the host and the vector (Siebers et al., 2016). In phytopathogenic bacteria, different types of lipids can determine compatibility with the host. Numerous research papers describe the role of FAs as diffusible signal factors (DSF) acting as modulators of different pathways in cell-to-cell communication to modulate QS and virulence (Wang and Qian, 2019). In phytopathogenic bacteria such as *X. fastidiosa* responsive of olive quick decline syndrome (OQDS), DSF-based QS model promotes biofilm formation and stickiness, determining (a) degradation of pit membranes to enable cross-vessel diffusion in the xylem; (b) twitching motility of bacterial cells; and (c) adhesion to the xylem surface and the switch from the planktonic endophytic lifestyle to the sessile insect-acquisition stage (Chatterjee et al., 2008; Beaulieu et al., 2013; Ionescu et al., 2016). The QS regulation is based on a delicate balance of several DSFs [e.g., MUFAs: lauroleic acid (C12:1), myristoleic acid (14:1), palmitoleic acid (16:1), oleic acid (18:1)] (Lindow et al., 2014; Ionescu et al., 2016).

As reported in Figure 1, oleic acid is the major substrate for the DOX-mediated synthesis of 10-HPOME and 7,10-diHOME (Martínez and Campos-Gómez, 2016). The role of oxylipins in mediating autocrine or paracrine signaling in communication is reported in several recent papers (Martínez et al., 2019; Niu et al., 2020; Scala et al., 2020). Despite their importance in eukaryotes and in plant–fungi interactions, the role of oxylipins is overlooked in phytopathogenic bacteria. Only recently, Martinez and colleagues demonstrate that unsaturated FAs can act as substrates for oxidizing enzymes (e.g., LOX and DOX) to form oxylipins that, in the opportunistic bacterial pathogen *Pseudomonas aeruginosa*, may transform into mono- and di-hydroxylated derivatives during the interaction with the host (e.g., *Drosophila*, lettuce) (Martínez and Campos-Gómez, 2016).

### Enzymatic biosynthesis of oxylipins

#### LOX-derived oxylipins in bacteria

Lipoxygenase LOXA, which was first identified in the human parasite *P. aeruginosa*, was the first prokaryotic lipoxygenase ever to be characterized. LOXA transforms arachidonic acid into 15-hydroxyheicosatetraenoic acid (15-HETE) (Vance et al., 2004). LOXA is secreted by *P. aeruginosa* in the lungs and oxidizes the PUFAs. Several biological roles, including interference with the host lipid signaling, and modulation of bacterial invasion have been hypothesized (Morello et al., 2019).

In liquid culture, *X. fastidiosa* accumulates different oxylipins, in particular, 10-HPOME, 10-HOME, and epoxyoctadecamonoenoic acids (EpOMEs), with different intra/extra-cellular distribution. LOX enzyme-derived oxylipins (i.e., 13-HODE, 9-HODE; 8,13-diHODE, 13HOTrE, and methyl jasmonic acid) are less represented (Christensen and Kolomiets, 2011). The plant stress hormone methyl jasmonate is secreted by *X. fastidiosa in vitro* and, overall, in large amounts in artificially infected plant tissues (Nomura et al., 2005). These results indicate that *X. fastidiosa* can synthesize and secrete oxylipins suggesting that, although oxygenation may occur inside the cell, oxylipins are transported through the outer membrane and accumulate in the medium (Martínez and Campos-Gómez, 2016; Scala et al., 2018). Recent *in vitro* studies on *X. fastidiosa* subsp. *pauca* demonstrate that the 9-LOX-derived oxylipins promote biofilming, whereas DOX-derived oxylipins stimulate planktonic growth and inhibit biofilm formation (Scala et al., 2020).

#### DOX-derived oxylipins in bacteria

In *P. aeruginosa*, DOXs including diol synthase catalyze the stereospecific oxygenation of oleic acid (Martínez and Campos-Gómez, 2016) to synthesize 10-HOME and 7,10-diHOME. These oleic acid-derived oxylipins are involved in regulating motility, biofilm formation, and virulence. In the QS regulating system, they inhibit type III pili-induced-motility by stimulating the expression of type IV pili, thus promoting twitching and aggregation in micro-colonies and biofilm formation, *in vitro*. These oxylipins are promoters of virulence on *Drosophila melanogaster* and lettuce (Martínez and Campos-Gómez, 2016). *P. aeruginosa* can co-opt host oxylipins to let them operate as environment-specific QS signals. A recent study (Martínez et al., 2019) highlighted a new oxylipin-dependent quorum sensing system (ODS) and the role of oxylipins produced from the host's oleic acid as auto-inducers of lifestyle switch in *P. aeruginosa* (Martínez et al., 2019).

Research on *X. fastidiosa* highlights similar trends (Scala et al., 2018, 2020), suggesting that oxylipins' role in autocrine bacterial cell communication (Niu et al., 2020) could be common across bacterial families (Martínez et al., 2019).

### Oxylipin signaling in plant–bacteria interactions

Although studies on oxylipins in plant–bacteria interactions are few and primarily focused plant oxylipins, nevertheless, a pivotal study by Martínez and Campos-Gómez (2016) suggests that oxylipins may also be crucial in bacterial communication. This section focuses on several recent studies that suggest that bacterial oxylipins may also be important signals in the intricate signaling network between plant hosts and pathogenic bacteria.

The example of *Arabidopsis thaliana* inoculated with *Pseudomonas syringae* pv. *tomato* (Pto) represents one of the first studies based on the cross talk mediated by the oxylipins during plant–bacteria interaction. The pre-treatment of plant with 9-LOX- and α-DOX-oxylipins from linoleic acid activated SAR and protected plant tissues against infection through a JA-independent signaling pathway (Hamberg et al., 2003; Prost et al., 2005; Truman et al., 2007; Vellosillo et al., 2007; Jung et al., 2009; Xia et al., 2009; Chanda et al., 2011; Vincent et al., 2012). In plant, the presence of 9-LOX-derived oxylipins induces brassinosteroid synthesis (hormones important for plant development and growth), SAR, and cell wall-based defense such as callose deposition (Hamberg et al., 2003; Vellosillo et al., 2007; Marcos et al., 2015). Among 9-oxylipins, 9-ketooctadecatrienoic acid pre-treatment modifies hormone homeostasis during infection and interferes with the hormonal changes caused by bacterial effectors (Vellosillo et al., 2007). JA, generated within 13-LOX pathway of linolenic acid, participates in the establishment of SAR after infection by biotrophic bacteria (Truman et al., 2007). 13-LOX derivatives participate in plant defense as regulators of gene expression, cell death, and antimicrobials (Stintzi, 2000; Vollenweider et al., 2000; Montillet et al., 2004; Prost et al., 2005).

**Figure 3:**
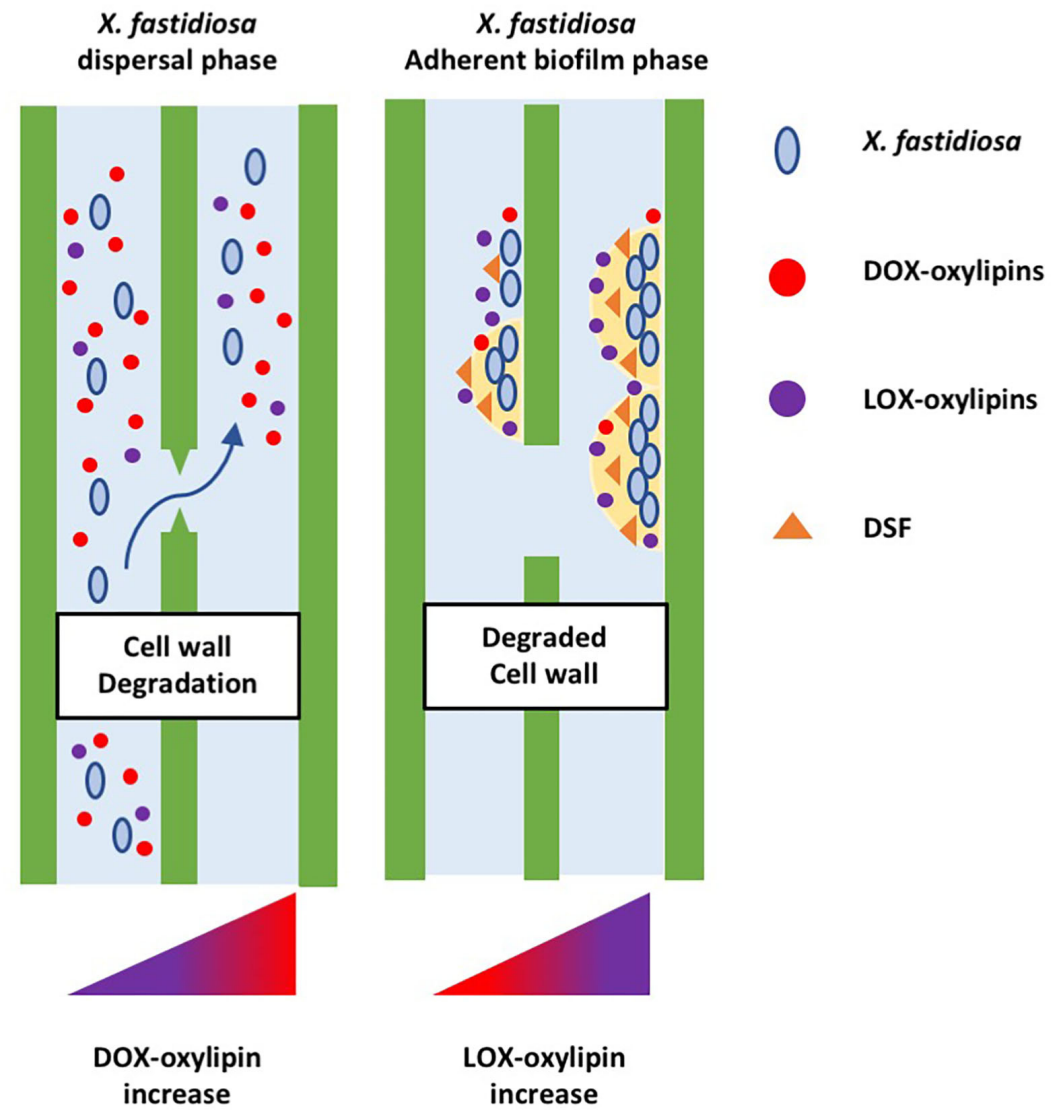
Hypothetical model involving oxylipins as signals in *X. fastidiosa–*host interaction. At the begin of host–bacteria interaction, the host does not recognize the presence of the pathogen, the bacterium modulates the planktonic-biofilm by itself, thought DFS-QS and ODS, and accumulates mainly DOX-oxylipins. Later, the plant recognizes the pathogen, activates the defense response, and triggers plant LOX-oxylipins. The bacterial pathogen switches in the “acquisition phase” by DSF-QS and ODS, accumulating mainly LOX-oxylipins. The bacterium–host interaction stimulates the bacteria biofilming, vector acquisition, and extensive vascular blockage plants, favored by a LOX oxylipins pathway in response to as an adaptive strategy to cope with harsh environmental conditions and to establish pathogenic insult with their host.

Details of the oxylipin-mediated communication between plant and phytopathogenic bacteria were provided by several studies on *X. fastidiosa*. The infection caused by *X. fastidiosa* was evaluated in plant model systems. *A. thaliana* induces the expression of ethylene/JA pathway to limit oxidative damage and represses the salicylic acid pathway (Rogers, 2012; Pereira et al., 2019); *Nicotiana tabacum* leads to a differential accumulation of specific lipid entities, including the oxylipins (Scala et al., 2018). Oxylipins emerged as hallmarks of pathogenic invasion in host tissues: Plants infected with *X. fastidiosa* accumulate oleic acid- and linoleic acid-derived oxylipins (e.g., 7,10-diHOME and 13-HODE). The study of oxylipin metabolism in the *X. fastidiosa* pathosystem was investigated also in *Olea europaea*. Symptomatic trees of the susceptible olive cultivar Ogliarola salentina accumulate ten lipid compounds that should be defined as hallmarks of OQDS (Scala et al., 2019). Identified hallmarks include 10-HOME (DOX-oxylipins); oleic and linoleic acid; LOX-derived 9- and 13-oxylipins (9-HODE, 9-OXODE, 9-HOTRE, 13-HODE, 13-OXODE, and 13-HOTRE). All the identified compounds were tested *in vitro* to unveil their effect on planktonic or biofilming state of *X. fastidiosa* subsp. *pauca*. The 7,10-diHOME, a DOX-derived oxylipin, downregulates biofilm formation, while LOX oxylipins from linoleic acid stimulate it. Recently, combined mass spectrometry/machine learning approach demonstrated that 13-HODE is a hallmark of OQDS and a susceptibility factor for olive tree toward *X. fastidiosa* (Scala et al., 2022). Considering the lipidome profile of the bacteria alone, the host–pathogen pathosystem, and available literature (Chatterjee et al., 2008; Rapicavoli et al., 2018; Roper et al., 2019; Scala et al., 2020), we argue that in plant tissue infected by *X. fastidiosa*, oxylipin involvement occurs from the early stage of infection just before host–pathogen recognition. At this stage, the pathogen modulates the planktonic-biofilm through DFS-QS and ODS; DOX-oxylipins are mainly accumulated for extensive vessel colonization, leading the planktonic state. At a later stage of infection, the host identifies the pathogen, activates the defense response, and triggers plant LOX-oxylipins. The accumulation of LOX oxylipins leads to a switch to the “acquisition phase” in the pathogen, a step of the infection cycle that culminates with the bacteria acquisition from the xylem sap to the insect vector (Figure 3). LOX accumulation is induced by the bacterium–host recognition and stimulates biofilm formation, vector acquisition, and extensive vascular blockage in plants, in accordance with the results obtained in the *A. thaliana*-Pto interaction. The activation of the LOX oxylipin pathway, in response to the plant–pathogen interaction, seems to be an adaptive strategy for the bacteria to cope with harsh environmental conditions and establish pathogenic insult with the host (Mosblech et al., 2009; Wasternack, 2014). A reversible cycle is *de facto* in place among the dual state of bacteria up to the host immune-system activation. Arguably, the pathogen regulates its behavior through FAs (as the DSFs) and oxylipins to trigger systemic invasion, limiting biofilm formation and acquisition by insect vectors.

These studies suggest that oxylipins have a significant role in determining the fate of the interactions: Resistance *vs*. susceptibility and that plant on one side and bacteria on the other try to adapt to the oxylipin signature formed during their interaction to exploit the weaknesses of the opponent.

## Conclusion

Lipid-mediated signal communication is an issue ongoing with numerous implications in plant–fungi and plant–bacteria disease control. This review focused primarily on oxidized lipids, the oxylipins, implicated in host–pathogen interactions, notably when the plant is exposed to fungal or bacterial disease. In plants, the oxylipins—jasmonates apart—are mainly involved in the defense against pathogens; in pathogens, a dual role emerges: oxylipins are produced as autocrine signals to mediate the development and differentiation (i.e., sexual sporulation in fungi; phase transition in bacteria), and paracrine signals to entertain a communication during plant diseases. The LOX pathway is the best characterized in plants and seems to be crucial for communicating with both fungi and bacteria, probably antagonizing and modulating the pathogen's response. LDS pathway seems to be crucial for the pathogen invasion (i.e., fungi and bacteria). The identification of oxylipins common or structurally similar in plants, fungi, and bacteria consolidates the theory of cross-kingdom communication. We can suggest that the ability to “recognize and react” to specific oxylipins may drive the fate of the interaction for the host: susceptibility vs. resistance.

Despite numerous findings regarding the specific oxylipins that are exchanged during the interactions, many questions remain open. One of these concerns the understanding of the receptor and transduction system of oxylipins that remains still unclear.

## Author contributions

MB, MR, and VS: conceptualization. MSa, MB, and NP: methodology. MR, MSc, and SL: investigation and funding acquisition. MB and VS: writing—original draft. MB, VS, BM, MZ, MSc, NP, and SL: writing—reviewing and editing. MB: visualization. MR and VS: supervision. All authors contributed to the article and approved the submitted version.

## Funding

This study was funded by MIPAAFT, Project Oli.Di.X.I.It (Olive growing and defense against Xylella fastidiosa and vector insects in Italy), D.M. 23773 of 6/09/2017, Project SALVAOLIVI (Safeguard and enhancement of the Italian olive-growing heritage with research actions in the phytosanitary defense sector), D.M.33437 of 12/21/20. MIUR National Operational Program Research and Innovation 2014-2020 (PON RI 2014-2020) Development of Nutraceuticals from Natural Sources - ARS01_01166. PUT National Operational Program “Enterprises and Competitiveness” 2014–2020 ERDF, UR - Development of new technologies in precision agriculture for the sustainable production of potato genotypes with high nutritional qualities (Acronym SOS TATA), no. F / 200088 / 01-03 / X45.

## Conflict of interest

The authors declare that the research was conducted in the absence of any commercial or financial relationships that could be construed as a potential conflict of interest.

## Publisher's note

All claims expressed in this article are solely those of the authors and do not necessarily represent those of their affiliated organizations, or those of the publisher, the editors and the reviewers. Any product that may be evaluated in this article, or claim that may be made by its manufacturer, is not guaranteed or endorsed by the publisher.

## Abbreviations

12-OH-JA: 12-hydroxy jasmonic acid
COX: cyclooxygenase
CYP450: cytochrome P450
DOX: dioxygenase
FA: fatty acid
GPCR: G protein-coupled receptors
HR: hypersensitive response
ISR: induced systemic resistance
JA: jasmonic acid
LDS: linoleate diol synthase
LOX: lipoxygenase
MUFA: monounsaturated fatty acid
ODS: oxylipin-dependent quorum sensing system
OQDS: olive quick decline syndrome
PTO: *Pseudomonas syringae* pathovar tomato
PUFA: polyunsaturated fatty acid
QS: quorum sensing
ROS: reactive oxygen species
SAR: systemic acquired resistance.
